# Effects of Janus Kinase Inhibitors on Cardio-Vascular Risk in Rheumatic Diseases: A Prospective Pilot Study

**DOI:** 10.3390/jcm14134676

**Published:** 2025-07-02

**Authors:** Diana Popescu, Minerva Codruta Badescu, Elena Rezus, Daniela Maria Tanase, Anca Ouatu, Nicoleta Dima, Oana-Nicoleta Buliga-Finis, Evelina Maria Gosav, Ciprian Rezus

**Affiliations:** 1 Department of Internal Medicine, Grigore T. Popa University of Medicine and Pharmacy, 16 University Street, 700115 Iasi, Romania; dr.popescu.diana@gmail.com (D.P.); daniela.tanase@umfiasi.ro (D.M.T.); anca.ouatu@umfiasi.ro (A.O.); nicoleta.dima@umfiasi.ro (N.D.); oana-nicoleta.buliga-finis@umfiasi.ro (O.-N.B.-F.); evelina.maria.gosav@umfiasi.ro (E.M.G.); ciprian.rezus@umfiasi.ro (C.R.); 2 3rd Internal Medicine Clinic, “Saint Spiridon” County Emergency Clinical Hospital, 700111 Iasi, Romania; 3 Department of Rheumatology and Physiotherapy, Grigore T. Popa University of Medicine and Pharmacy, 16 University Street, 700115 Iasi, Romania; elena.rezus@umfiasi.ro; 4 Rheumatology Clinic, Clinical Rehabilitation Hospital, 700661 Iasi, Romania

**Keywords:** rheumatic disease, rheumatoid arthritis, atherosclerosis, Janus kinase inhibitors, carotid intima-media thickness, subclinical vascular disease, lipoprotein(a)

## Abstract

**Background/Objectives:** Patients with rheumatoid arthritis (RA) and psoriatic arthritis (PsA) exhibit increased cardiovascular risk, partly attributed to persistent systemic inflammation. Janus kinase inhibitors (JAKi) effectively reduce inflammation, but their impact on cardiovascular risk remains unclear. This pilot study aimed to evaluate the effect of JAKi therapy on systemic inflammation and lipid markers, correlate traditional cardiovascular risk factors with biological parameters, and quantify subclinical atherosclerosis progression. **Methods**: We conducted a prospective, single-center study including 48 patients receiving JAKi. Clinical, inflammatory, lipid, and vascular parameters were assessed at baseline (T0) and after 12 months (T1). Primary endpoints included changes in carotid intima-media thickness (cIMT), ankle-brachial index (ABI), and carotid plaque presence. **Results:** Mean cIMT significantly decreased from 0.29 mm to 0.125 mm (*p* = 0.019), while ABI improved modestly, but not significantly (0.125 to 0.04, *p* = 0.103). Carotid plaque prevalence increased slightly from 39.6% to 47.9%, *p* = 0.159. C-reactive protein (CRP) levels declined significantly, while interleukin (IL)-1β levels increased. Lipoprotein(a) [Lp(a)] levels decreased significantly (mean reduction −7.96 mmol/L, *p* = 0.001). Multivariate regression identified Lp(a) as an independent predictor of carotid plaque at both T0 (*p* = 0.011) and T1 (*p* = 0.005). Baseline ABI was a significant predictor of acute cardiovascular events [hazard ratio (HR): 4.614, 95% CI: 1.034–20.596, *p* = 0.045]. **Conclusions**: JAKi therapy significantly reduced systemic inflammation and cIMT in patients with autoimmune rheumatic diseases, suggesting a potential benefit in attenuating early vascular changes. However, residual cardiovascular risk remains in patients with low ABI and elevated Lp(a), warranting close monitoring.

## 1. Introduction

Patients with chronic immune-mediated rheumatic diseases, such as rheumatoid arthritis (RA) and psoriatic arthritis (PsA) experience significantly elevated cardiovascular morbidity and mortality, with estimates suggesting a 1.5 to 2-fold increased risk of major adverse cardiovascular events (MACE) compared to the general population of similar age and sex [1,2,3]. The presence of traditional cardiovascular risk factors alone cannot explain such an increased risk [4]. Therefore, it was hypothesized that chronic systemic inflammation plays a central role in promoting atherosclerosis, vascular dysfunction, and arterial remodeling in these patients [5,6].

Subclinical vascular damage precedes overt cardiovascular disease and often remains undiagnosed until late stages [7,8]. Therefore, non-invasive tools to evaluate early vascular alterations are crucial in patients with rheumatic conditions. Among these, the carotid intima-media thickness (cIMT) and the presence of carotid atheromatous plaques, both measurable by high-resolution ultrasound, have been established as reliable markers of subclinical atherosclerosis and predictors of future cardiovascular events [9,10]. Additionally, ankle-brachial index (ABI) provides insight into peripheral arterial disease (PAD) and systemic atherosclerotic burden [11,12,13]. Monitoring these parameters allows early identification of vascular involvement and implementation of therapeutic interventions to modulate cardiovascular risk.

JAKi are targeted synthetic DMARDs (tsDMARDs), acting against receptor-associated tyrosine kinases. These small-molecule inhibitors specifically target the JAK-STAT signaling pathway, which plays a crucial role in mediating immune responses and inflammation [14,15]. Clinical trials and real-life data continue to support the use of JAKi in patients who have not responded well to traditional therapies, providing a valuable option, especially for those resistant to biologic drugs, such as TNF inhibitors [16,17]. Importantly, the cardiovascular safety of JAKi has recently come under scrutiny following the ORAL Surveillance trial, which reported an increased risk of major adverse cardiovascular events with tofacitinib compared to TNF inhibitors in patients with RA and cardiovascular risk factors [18]. These findings prompted regulatory agencies to issue safety warnings and update prescribing guidelines. However, real-life data remain scarce, and the extrapolation of findings from selected high-risk populations to broader clinical settings is challenging [19,20]. This underscores the urgent need for additional evidence regarding the vascular effects of JAKi in broader populations, especially using validated, non-invasive cardiovascular markers such as cIMT and ABI.

Despite the growing interest in the vascular effects of JAKi, data on their longitudinal impact on established vascular markers such as cIMT, ABI, and carotid plaque remain limited, particularly in real-life cohorts with autoimmune rheumatic diseases. Furthermore, the interplay between inflammatory markers, lipid parameters, and vascular outcomes remains incompletely elucidated. Our study addresses this gap by evaluating vascular outcomes and biomarker dynamics over a 12-month period in patients with RA or PsA treated with JAKi. Although guidelines have recommended restrictions on the use of JAKi in high-risk individuals, real-life data on vascular outcomes in such patients are extremely limited. Clinical trials have often excluded elderly patients or those with multiple comorbidities, making extrapolation to daily practice uncertain. Addressing this gap is especially relevant because, in real life, many patients eligible for JAKi have cardiovascular risk profiles that would have excluded them from the landmark trials.

In this context, our study aims to evaluate the effect of JAKi therapy on vascular parameters over a 12-month period and correlate cardiovascular biomarkers with subclinical vascular changes. We focused on non-invasive indicators of subclinical vascular damage and on relevant lipid and inflammatory biomarkers. The objective of the study was to determine whether 12 months of JAKi treatment could modulate atherosclerosis progression or vascular function, and to explore correlations between changes in vascular parameters and shifts in inflammation and lipid profile. By focusing on these endpoints, we aimed to gain a more accurate perspective on the potential cardiovascular benefits or risks of JAKi in rheumatic patients and to bridge the current knowledge gap regarding tsDMARDs and vascular health. We strongly believe that understanding these effects will help develop holistic management strategies that will reduce both the consequences of joint diseases and cardiovascular comorbidity in this category of patients.

## 2. Materials and Methods

### 2.1. Study Design and Ethical Approval

We conducted a prospective, single-center, analytical, and observational study. Between September 2022 and June 2024, we enrolled 59 adult patients previously diagnosed with RA or PsA based on international diagnostic criteria. All subjects provided written informed consent prior to inclusion in the study. The study was approved by the Ethics Committee of “Grigore T. Popa” University of Medicine and Pharmacy Iasi (No. 222/23.08.2022), “Saint Spiridon” County Emergency Clinical Hospital (No. 24/11.03.2022), and Clinical Rehabilitation Hospital Iasi (No. 08/22.06.2022). All procedures were conducted in accordance with the Declaration of Helsinki.

### 2.2. Patient Selection and Protocol

**Figure 1:**
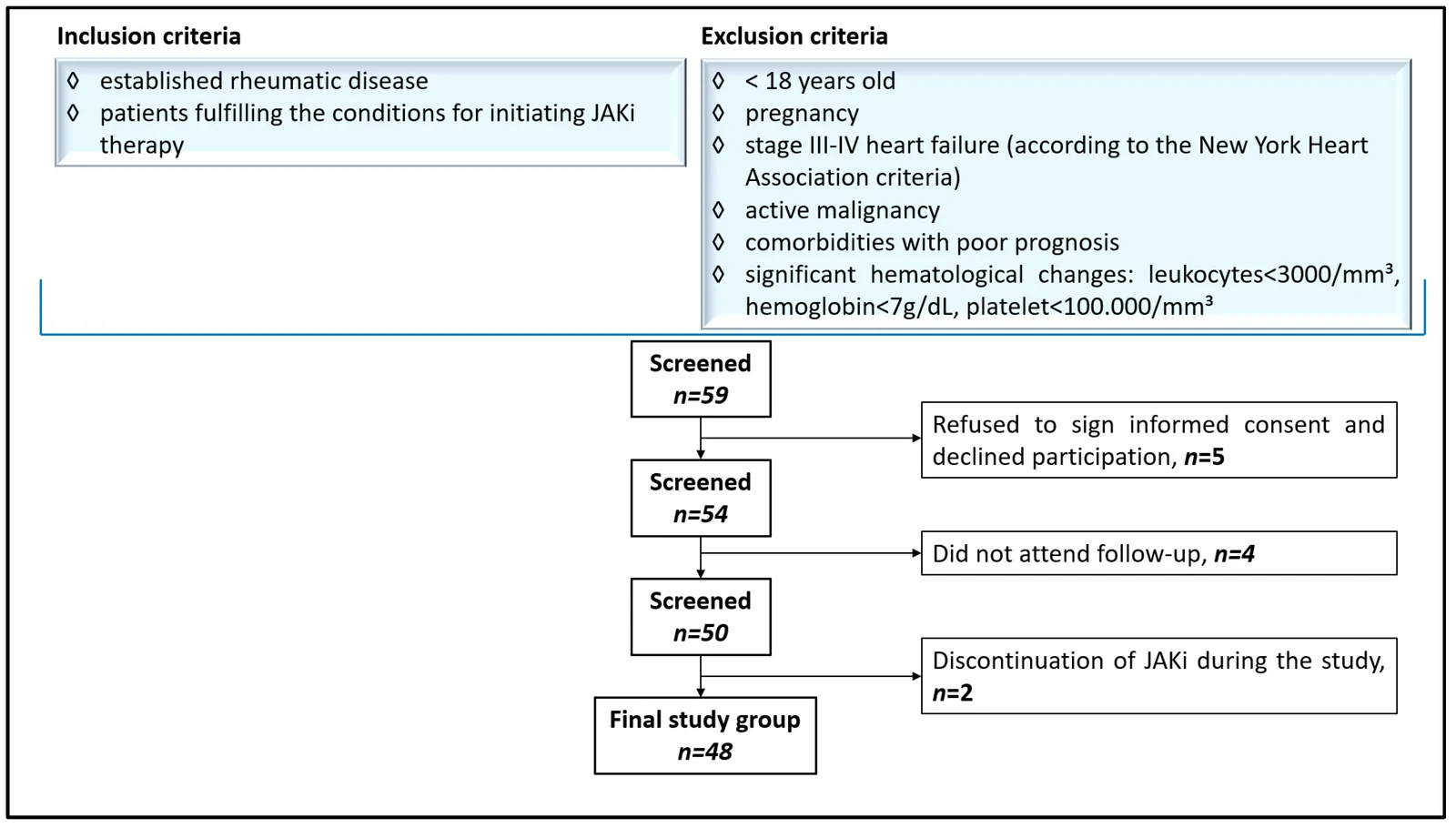
Cohort selection, and the inclusion and exclusion criteria (JAKi).

We assessed 59 Caucasian adult patients eligible for treatment with JAKi (baricitinib, tofacitinib, and upadacitinib) according to the recommendations of the European League Against Rheumatism (EULAR) and the American College of Rheumatology guidelines. Eligibility was not dependent on prior exposure to conventional or biological DMARDs, nor on the presence of cardiovascular risk factors or a personal history of cardiovascular disease. Patients were included if they were 18 years of age or older and met the criteria for initiating biological therapy. Of the initial 59 patients, 11 were excluded based on the protocol presented in Figure 1. In the end, 48 patients met the inclusion criteria and were enrolled in the final analysis.

Before initiating biological therapy, all patients underwent a comprehensive clinical evaluation. We collected data on traditional cardiovascular risk factors, such as age, sex, and smoking status, and recorded detailed medical history and current medication. The main comorbidities searched for included arterial hypertension, dyslipidemia, diabetes mellitus (DM), and PAD. History of acute cardiovascular events such as myocardial infarction (MI), unstable angina, or stroke was also recorded. Medications of interest included conventional synthetic DMARDs (csDMARDs) and cardiovascular-related drugs, such as lipid-lowering agents, antihypertensives, antiplatelet agents, and anticoagulants. Additionally, disease characteristics were recorded, including the presence of rheumatoid factor (RF) and anti-citrullinated protein antibodies (ACPA).

The clinical examination included the assessment of anthropometric parameters [height (m), weight (kg)], with the calculation of body mass index (BMI) (kg/m^2^). The normal range was defined as 18.5–25 kg/m^2^. Cardiovascular evaluation included examination of the heart and peripheral arteries. We evaluated the presence of heart murmurs, peripheral pulses, heart rate and rhythm, and measured blood pressure (BP). High BP was defined according to the current European Society of Cardiology (ESC) Guidelines for the management of elevated blood pressure and hypertension as systolic BP ≥ 140 mmHg and/or diastolic BP ≥ 90 mmHg [21]. To non-invasively assess atherosclerotic vascular damage, we calculated the ABI by dividing the systolic BP at the ankle by the systolic BP at the arm. The normal ABI range was defined as 1.0 to 1.4, with values below 0.9 indicating the presence of PAD. An ABI greater than 1.4 may suggest stiffened arteries due to calcification, often seen in patients with DM or chronic kidney disease (CKD), and may require further investigation.

All patients were examined by the same medical team, which included the PhD student and the supervising physician, both specialists in internal medicine.

### 2.3. Study Procedures

An extensive paraclinical assessment was performed for each patient prior to the initiation of JAKi therapy and at the 12-month follow-up. We designated the initial assessment as the evaluation at time 0 (T0) and the reassessment after 12 months of treatment as time 1 (T1).

Blood samples were used to evaluate hematological parameters, inflammatory status, and lipid profile. Dyslipidemia was diagnosed if any of the following was present: elevated total cholesterol (TC > 200 mg/dL), elevated low-density lipoprotein cholesterol (LDL-C > 130 mg/dL), elevated triglycerides (TG > 150 mg/dL), or elevated lipoprotein a (Lp(a) > 75 mmol/L). Based on previous studies indicating that low-to-moderate doses of statins typically lower LDL-cholesterol by 30% [22], a significant reduction in LDL-C was defined as a decrease of ≥30%. The inflammatory status was evidenced by increased levels of C-reactive protein (CRP > 0.5 mg/dL), IL-6 (>7 pg/mL), or IL-1β (>5 pg/mL).

Carotid ultrasound examination was used to assess the presence of atheromatous plaque, defined as a focal thickening of the arterial wall protruding into the lumen by at least 0.5 mm or by 50% more than the surrounding vessel wall. The main variable assessed was cIMT, measured using B-mode ultrasound by a single experienced radiologist with the same ultrasound equipment. cIMT was calculated from longitudinal views, 1–2 cm below the bifurcation, by measuring the distance between the inner (intima) and outer (media) layers of the arterial wall. A cIMT value greater than 1.2 mm was considered abnormal and was associated with an increased risk of acute cardiovascular events [23].

### 2.4. Statistical Analysis

A preliminary descriptive analysis was carried out to examine the dataset. Qualitative variables were presented as counts and percentages, while quantitative data were processed using the Statistical Package for Social Sciences, Chicago, Illinois (SPSS) software, version 23.0. For continuous variables with a normal distribution, results were expressed as means ± standard deviation (SD); for those lacking normal distribution, medians and interquartile ranges were reported. Frequencies and percentages were used for both categorical and continuous variables where appropriate. The Chi-square test was utilized for analyzing categorical variables.

To explore the relationships between variables, the Spearman correlation test was applied. A *p*-value of ≤0.05 was considered statistically significant for all tests. Additionally, the Spearman correction was applied to the analysis of clinical and laboratory tests, including IL-6, IL-1β, and biological therapy, as well as dynamic assessments.

Univariate and multivariate Cox Proportional Hazard Models were employed to predict outcomes in patients with rheumatic diseases and cardiovascular risk factors, incorporating both time-based data, biological treatment, and demographic variables.

## 3. Results

### 3.1. Study Characteristics, Clinical and Biological Assessment

Forty-eight adult patients with rheumatic diseases treated with JAKi completed the study and were included in data analysis. The number of patients receiving baricitinib, upadacitinib, and tofacitinib was 20, 15, and 13, respectively. Out of 48 patients, 46 were diagnosed with RA, most of them with positive RA (*n* = 35; 76%) and positive ACPA (*n* = 36; 78%), and only 2 with PsA.

The demographic analysis identified 39 women (83.3%) and 9 men (16.7%) in the study group, with an average age of 56.83 years. Of the participants, 25% were under 50 years old, while the remaining 75% were over 50, with none exceeding 75 years old. Selected baseline characteristics are detailed in Table 1. The histogram on the age distribution in the study group was homogenous, while the ratio of women to men was not homogenous, but without statistical significance (mean 0.17, SD 0.377).

Arterial hypertension was the most common comorbidity, affecting 28 patients (58%), followed by dyslipidemia in 17 patients (35.4%), of whom 12 were already undergoing lipid-lowering therapy. Nine patients had a prior diagnosis of DM (19%), while none had PAD. Additionally, 8 patients (17%) were active smokers, and 36 patients (75%) had an elevated BMI. One patient has a history of stroke, and none of MI or venous thromboembolism.

Various general and clinical parameters were analyzed statistically. Normality tests, including the One-Sample Kolmogorov-Smirnov Test, were conducted for each sex, and an overall score was calculated. Results indicated a normal distribution for arterial hypertension and dyslipidemia, whereas other variables exhibited an abnormal distribution; however, these differences were not statistically significant. Spearman’s correlation coefficient was applied to all variables with a non-parametric distribution.

About half of our patients had elevated TC and LDL-C levels at baseline, with no significant changes after initiation of JAKi therapy. We found moderate correlations between dyslipidemia and other cardiovascular risk factors, such as arterial hypertension or DM.

When we assessed the inflammatory status, we found that 52% of patients had elevated CRP levels at baseline. After 12 months of JAKi therapy, this proportion decreased to 37.5% and the median CRP values dropped from 0.54 mg/dL to 0.25 mg/dL. The most pronounced CRP reduction was observed in patients aged over 50. For IL-1β, no patient had values above the normal threshold at baseline; however, at T1, 6 patients (12.5%) presented elevated levels. Additionally, minimal variation in IL-6 levels was observed at T1 compared to T0 (25% versus 23%).

### 3.2. Non-Invasive Vascular Assessment

The vascular status was evaluated using dynamic measurements of cIMT, ABI, and presence of atheromatous plaques at baseline and after one year of JAKi therapy.

At T0, 39.6% of patients presented atheromatous plaques, and 29.2% had elevated cIMT values. At T1, the proportion of patients with plaques increased to 47.9%, whereas elevated cIMT was observed in only 12.5% of patients. Interestingly, while the number of plaques increased, the prevalence of abnormal cIMT decreased. Similarly, at T0, 6 patients (12.5%) had reduced ABI, and this proportion fell to 2 patients (4.2%) at T1.

### 3.3. Correlations Between Vascular, Clinical, and Biological Parameters

Spearman’s correlation analysis revealed that arterial hypertension, dyslipidemia, and smoking were associated with subclinical atherosclerosis, although their influence diminished after initiating JAKi therapy. The correlation between arterial hypertension and the presence of atheromatous plaques declined from r = 0.425 at T0 to r = 0.303 at T1. Similarly, the association between smoking and ABI weakened from r = 0.338 at T0 to r = 0.187 at T1. However, a persistent moderate correlation between smoking and cIMT (r = 0.338 at both timepoints) suggests a sustained vascular impact of smoking, independent of therapy. Notably, the association between dyslipidemia and plaque presence was r = 0.192 at T0 and r = 0.415 at T1.

To identify independent predictors of vascular outcomes, multivariate linear regression models were applied at both baseline (T0) and 12-month follow-up (T1) for each vascular parameter: cIMT, ABI, and atheromatous carotid plaque presence. Key results of these analyses are summarized in Table 2, Table 3, Table 4 and Table 5.

The logistic regression models for carotid plaque presence demonstrated strong model fit at baseline and good fit at follow-up, indicating that the included predictors, particularly Lp(a), significantly contributed to explaining plaque presence.

To evaluate the impact of JAKi therapy on vascular parameters, we conducted a paired differential analysis comparing baseline and follow-up values.

We found that cIMT decreased significantly after treatment (mean difference: −0.165 mm, *p* = 0.019), pointing to a potential structural improvement in carotid artery walls. ABI also showed a downward trend. However, the change was not statistically significant (*p* = 0.103). The presence of carotid plaques increased slightly, but this change did not reach statistical significance (*p* = 0.159).

### 3.4. Risk of Acute Cardiovascular Events

**Figure 2:**
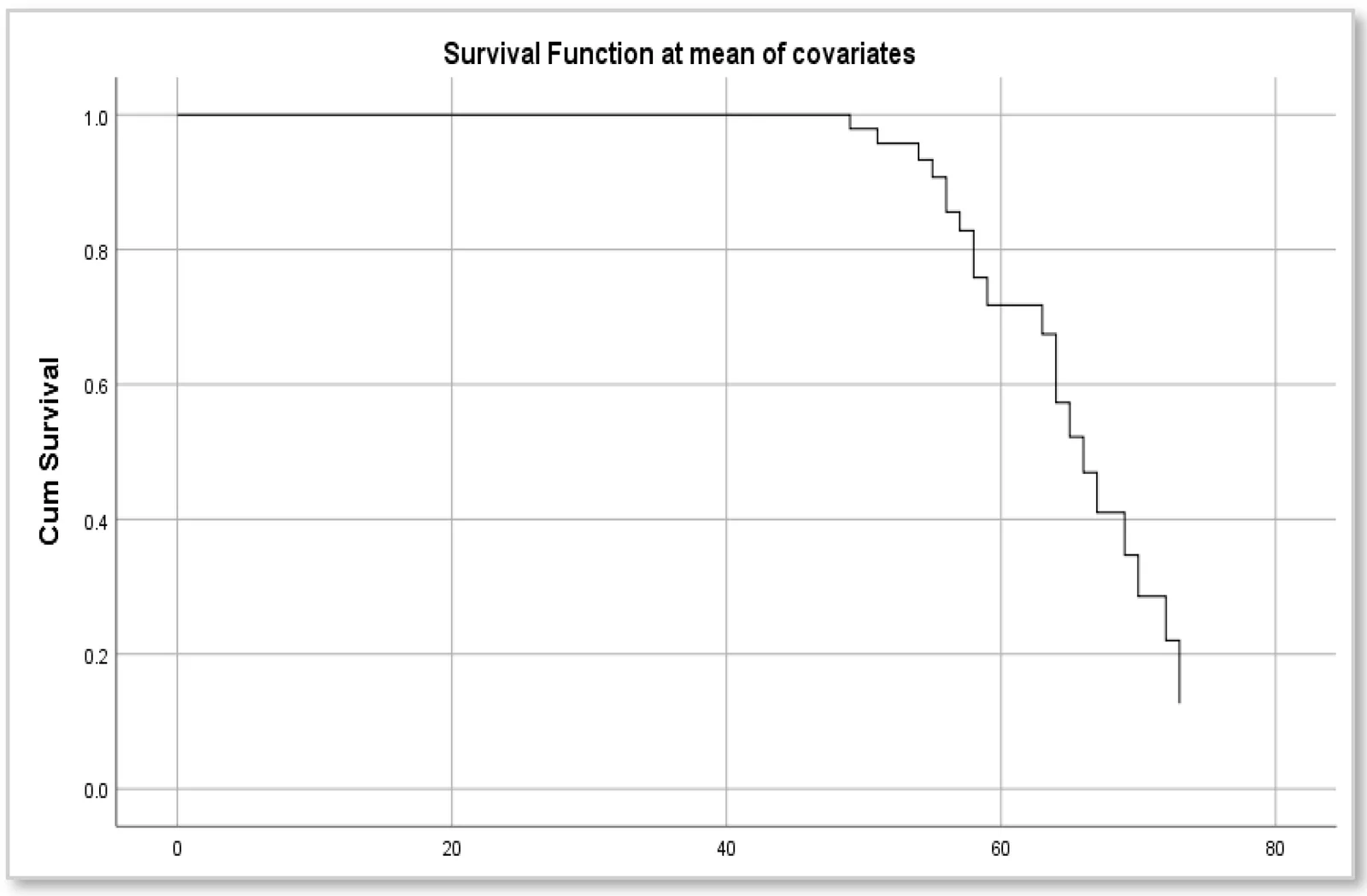
Kaplan-Meier Survival Curve stratified by baseline ABI and CRP levels in patients receiving JAKi.

A univariate Cox regression analysis evaluated the relationship between baseline vascular status parameters and the risk of acute cardiovascular events, considering elevated CRP levels. Among the vascular parameters analyzed, only the ABI at baseline (T0) demonstrated a statistically significant association with acute cardiovascular events, presenting a HR of 4.614 (95% CI: 1.034–20.596, *p* = 0.045). Patients exhibiting pathological ABI values before starting JAKi therapy had approximately a 4.6-fold increased risk of acute cardiovascular events compared to those with normal ABI. Kaplan-Meier survival curves illustrated a marked decrease in survival rates after age 50 among patients with abnormal baseline ABI and elevated CRP prior to JAKi therapy, highlighting the significant impact of early vascular dysfunction on long-term survival (Figure 2).

**Figure 3:**
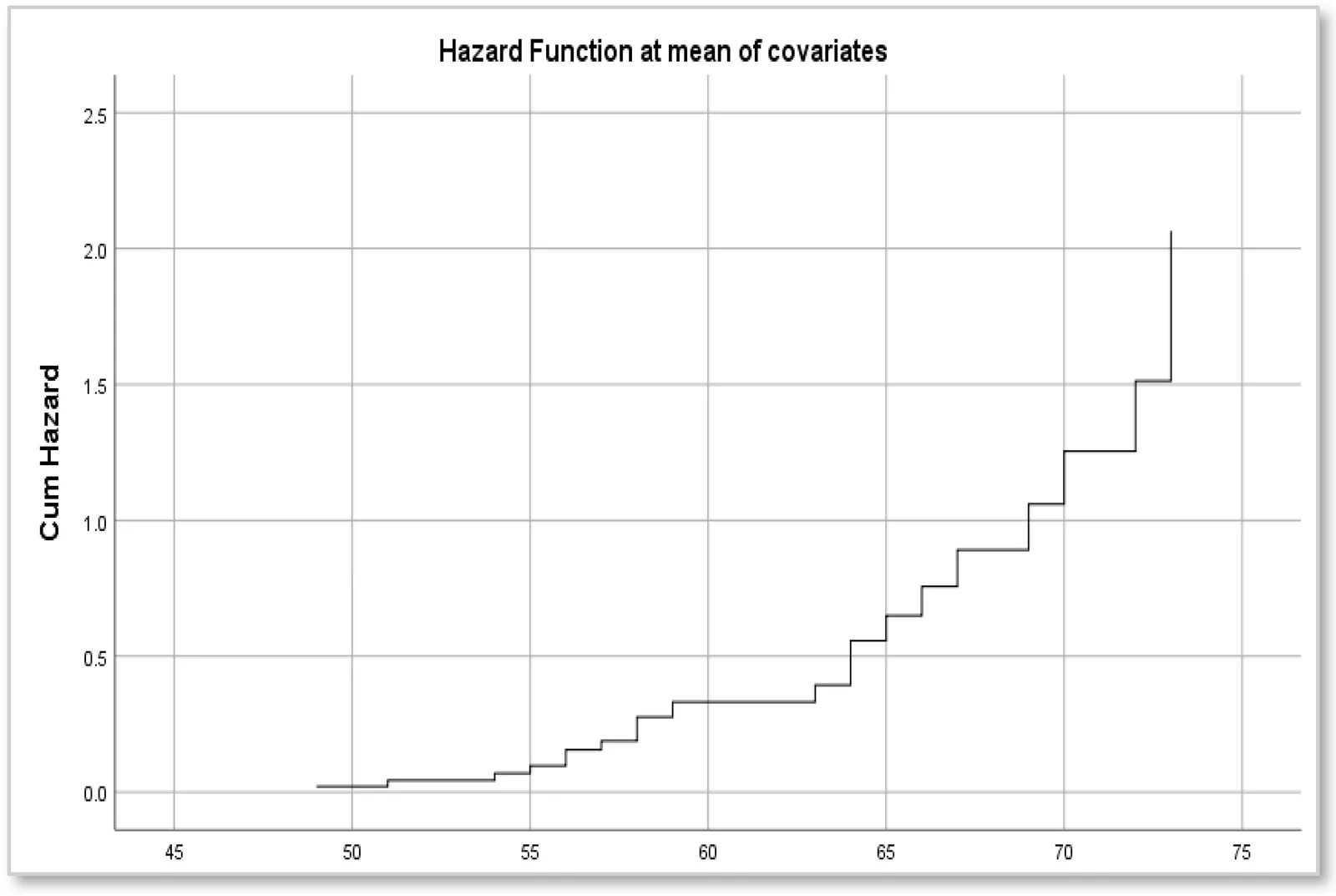
Age-related incidence of acute cardiovascular events in high-risk patients (abnormal ABI and CRP baseline levels).

Similarly, the incidence of acute cardiovascular events increased sharply after age 55 in this high-risk patient subgroup (Figure 3).

## 4. Discussion

The strength of our study is that it provides real-life evidence that JAKi therapy favorably modulates vascular parameters and subclinical atherosclerosis in patients with autoimmune rheumatic diseases. Our data illustrate the fine interplay between lipid metabolism, systemic inflammation, and subclinical vascular changes during JAKi therapy.

After 12 months of JAKi therapy, there was a significant reduction in systemic inflammation, illustrated by a drop in CRP, along with stabilization of surrogate markers of subclinical atherosclerosis. Specifically, mean cIMT significantly decreased over the course of follow-up, suggesting slowing of carotid atherosclerosis progression under JAKi therapy or even its partial reversal. This finding is noteworthy given that patients with RA or PsA are at approximately twice the cardiovascular risk of the general population due to chronic inflammation-driven accelerated atherosclerosis [1,24]. By effectively dampening pro-inflammatory cytokine signaling, JAK inhibition appears to partially alleviate the vascular damage. This aligns with the paradigm that rigorous inflammation control should be the central strategy for reducing cardiovascular risk in rheumatic patients [25]. The reduction in cIMT during the 12-month follow-up is due to the slowing of carotid artery wall thickening, which essentially represents a stabilization or slight regression of subclinical carotid atherosclerosis. These findings provide new evidence that cytokine inhibition has a positive impact on certain early markers of atherosclerosis in RA/PsA. Our data reinforce existing evidence suggesting that anti-inflammatory therapy slows vascular damage [26].

Notably, our results of cIMT regression with JAKi extend prior observations with other DMARDs. It was reported that patients receiving TNF-α blockers have smaller cIMT values and slower atherosclerosis progression compared to those on conventional therapy [27]. When comparing the vascular effect of various biologic therapies, it was found that patients treated with JAKi had significantly lower cIMT values than those on other therapies, including IL-6 blockers. Interestingly, this reduction in cIMT occurred despite the absence of significant differences in traditional cardiovascular risk factors or disease activity between the study groups. Thus, it was hypothesized that JAK inhibition has a direct vascular effect [27]. However, a short-term study of JAK inhibition in RA found no measurable change in carotid atherosclerosis or arterial stiffness at 3-month follow-up [28], indicating that longer-term suppression of inflammation may be required to influence structural vascular changes. Our 12-month data support this hypothesis. Sustained JAKi treatment can lead to favorable remodeling of the carotid artery wall, possibly by allowing time for arterial healing and reversal of inflammatory intimal thickening once the chronic immune assault is dampened.

The slight, statistically non-significant increase in carotid plaque prevalence in our cohort (from 39.6% to 47.9%) highlights the complexity of atherosclerotic disease in rheumatic patients. Plaque formation is a multifactorial and typically slow process. Even with improved inflammatory control, some patients developed new plaques or had progression of existing lesions over the course of a year. This highlights that residual cardiovascular risk persists despite immunosuppressive therapy. It is well-documented that RA patients can accumulate atherosclerotic plaques early in life, and the presence of carotid plaque confers a significantly elevated risk of cardiovascular events in this population [29]. While cIMT mainly reflects diffuse intimal thickening, which has a higher potential for reversibility, discrete plaques represent a more advanced focal disease that might not regress on the timescale of a year without aggressive risk factor modification. Our findings support the idea that controlling inflammation alone may not immediately shrink established plaques. Other studies reported similar results in patients with inflammatory arthritis, namely that despite effective anti-rheumatic therapy, plaque burden may remain unchanged in the medium term [27]. Longer observation or concomitant therapies, such as statins or antihypertensives, would be necessary to detect a decline in plaque number or size. Overall, the divergent behavior of cIMT versus plaque in our study suggests that cIMT may be a more responsive marker of early vascular improvement with anti-inflammatory treatment, whereas carotid plaques reflect a more entrenched pathology that requires prolonged time or additional interventions to influence.

These results reinforce the idea that JAK inhibition can modulate early vascular damage, although it may not completely halt the progression of atherosclerosis, especially in high-risk patients. These findings are consistent with previous studies focusing on vascular outcomes in JAKi-treated patients. Czókolyová et al. demonstrated that one year of tofacitinib therapy resulted in significant alterations in lipid fractions and adipokine levels, many of which correlated with vascular imaging markers such as pulse wave velocity (PWV), cIMT, and flow-mediated dilation (FMD). These results support the existence of a complex, yet balanced, cardiovascular effect of JAK inhibition in RA patients [30]. Furthermore, a prospective study design published by Anyfanti et al., which aimed to comprehensively evaluate the impact of JAKi on micro- and macrovascular parameters by measuring ambulatory BP, PWV, and using nailfold capillaroscopy, highlighted the multifactorial determinism of vascular risk modulation in JAKi-treated patients [31].

Our regression analyses shed light on the interplay between traditional cardiovascular risk factors and inflammation in driving vascular changes. At baseline, arterial hypertension and smoking, along with Lp(a), emerged as important predictors of carotid plaque presence, which is consistent with their well-known roles in atherosclerosis [28]. Interestingly, after 12 months of treatment with JAKi, arterial hypertension and smoking were no longer significant predictors in our models, while Lp(a) remained strongly predictive. This pattern could indicate that effective anti-inflammatory therapy might mitigate the impact of certain traditional cardiovascular risk factors on atherosclerosis progression. It was hypothesized that chronic systemic inflammation can amplify the deleterious vascular effects of risk factors, such as arterial hypertension. For instance, pro-inflammatory cytokines can impair endothelial function, making the endothelium more susceptible to hypertensive damage [32]. By quelling inflammation, JAKi might partially uncouple this synergy, rendering the vasculature more resilient to blood pressure control and smoking-related insults. We observed that blood pressure control and smoking status did not fully explain plaque outcomes once patients were on JAKi, hinting at a complex interaction between inflammation and traditional cardiovascular risk factors. Thus, this finding should be interpreted with caution, as it may reflect the limitations of our sample size and the relatively short follow-up period. Larger studies are needed to confirm whether inflammation reduction truly diminishes the contribution of traditional cardiovascular risk factors to cardiovascular risk in rheumatic disease patients. What remains indisputable is that baseline arterial impairment was a warning sign: patients with pathological ABI or existing carotid plaques at baseline likely harbor significant atherosclerosis and thus, continued to be at elevated risk of future major cardiovascular events. In fact, during our study, those with low ABI values had a higher incidence of cardiovascular events, emphasizing that established atherosclerotic disease confers a risk beyond that which can be immediately normalized by anti-rheumatic therapy.

Our study also provides critical insights into the risk of acute cardiovascular events among patients with autoimmune rheumatic diseases receiving JAKi therapy. Using Cox regression analysis, we identified baseline ABI as a significant predictor of acute cardiovascular events. Specifically, patients with pathological ABI exhibited a notably increased HR for acute cardiovascular events compared to those with normal ABI values. This substantial increase in risk emphasizes the importance of early detection of PAD as a prognostic marker of severe cardiovascular complications in rheumatic patients. These findings are consistent with previous studies demonstrating ABI’s predictive value for cardiovascular morbidity and mortality [33]. Low ABI values, reflecting significant peripheral atherosclerosis and arterial stiffness, have been correlated with increased cardiovascular risk, notably MI and stroke, in both rheumatic and non-rheumatic populations [33,34,35]. Our Kaplan-Meier survival analysis further emphasized the detrimental impact of impaired ABI combined with elevated baseline inflammatory markers, such as CRP. We observed a stark decline in event-free survival rates after age 50 in patients who presented both abnormal ABI and high CRP levels before initiating JAKi therapy. The incidence of acute cardiovascular events sharply rose after age 55 within this subgroup, highlighting a synergistic interplay between vascular impairment and systemic inflammation. Previous research, including the analysis by Rojas-Gimenez et al., has highlighted this link between chronic inflammation, evidenced by persistent CRP elevation, and accelerated cardiovascular damage in RA [27]. Interestingly, although the treatment with JAKi significantly reduced systemic inflammation, it did not completely eliminate the cardiovascular risk related to baseline arterial impairment in our cohort. This residual risk phenomenon aligns with the concept of multifactorial cardiovascular pathology, suggesting that vascular damage accumulated before initiation of therapy may have a persistent impact on long-term cardiovascular outcomes despite subsequent control of inflammation. The observation that baseline ABI independently predicted acute events highlights the critical window for cardiovascular intervention, suggesting that earlier initiation of aggressive risk factor management and therapy might be necessary in these high-risk patients. Future prospective studies should explore whether targeted interventions aimed at normalizing ABI, such as lipid-lowering agents, antiplatelet therapy, or lifestyle modifications, can effectively mitigate this elevated cardiovascular risk among patients initiating JAKi therapy.

The 2023 EULAR recommends that JAKi should be used in patients with cardiovascular risk factors only when there is no suitable alternative [36]. These recommendations are mainly based on the results of the ORAL Surveillance trial [18]. Notably, a significant proportion of patients in our cohort fell into the high-risk category: 75% were over 50 years of age, 58% had hypertension, 75% had an elevated BMI, and 17% were smokers. Despite this, our results showed a reduction in cIMT and a stabilization of inflammatory and lipid markers, supporting the vascular safety profile of JAKi in real-life practice. These findings are consistent with recent data suggesting that, with careful monitoring, JAKi may remain a viable option for certain high-risk patients [37,38]. Anyfanti et al. have highlighted the heterogeneity of vascular response in RA patients and the role of traditional cardiovascular risk factors, such as hypertension and obesity, in modulating the outcome of antirheumatic therapy [31]. Furthermore, a subsequent observational study by the same investigators assessed the effects of JAKi on coronary microvascular perfusion using the subendocardial viability ratio. Despite the presence of multiple cardiovascular risk factors in the patient cohort, no significant deterioration in myocardial perfusion was observed after three months of JAKi therapy [39]. Taken together, these data have important clinical implications for rheumatologists attempting to balance disease control and cardiovascular safety.

This study has several limitations that temper the strength of our conclusions. First, the sample size was limited, and there was no parallel control group of patients on alternative therapies for direct comparison. It remains possible that different RA treatments confer varying degrees of cardiovascular protection, as suggested by the superior cIMT profile observed with TNF inhibitors in cross-sectional analyses. Second, the 12-month follow-up, while practical, may still be too short to capture long-term vascular effects such as plaque progression /regression or clinical events. Subclinical parameters, such as cIMT, can require long durations to significantly change, and subtle improvements might have been missed without a larger sample. Lastly, by the nature of a real-life observational study, there may be confounding factors. For instance, patients with severe uncontrolled disease may have been preferentially started on JAKi, or conversely, those with significant baseline cardiovascular risk might have been steered toward other therapies due to JAKi safety concerns. We attempted to mitigate confounding through multivariate analyses and provided evidence that the reduction in inflammation was the key mediator of vascular outcomes. However, unmeasured factors could still play a role.

Using our results as a starting point, future research should aim to more conclusively determine the cardiovascular impact of JAKi in autoimmune rheumatic diseases. Larger, controlled trials are needed to validate our observations. Comparative studies between JAKi and other bDMARDs are needed to assess the differences in clinical cardiovascular outcomes. It would be especially informative to stratify patients according to baseline cardiovascular risk (high Lp(a), existing plaques, low ABI, etc.) to see if certain subgroups gain greater vascular benefit, or, conversely, face higher risk with JAKi. Additionally, another area for future research is the long-term trajectory of Lp(a) in patients on JAKi.

## 5. Conclusions

In conclusion, our study demonstrates that 12 months of JAKi therapy effectively reduces systemic inflammation and stabilizes surrogate markers of subclinical atherosclerosis in patients with autoimmune rheumatic diseases. Despite these beneficial effects, baseline arterial impairment, especially pathological ABI values, significantly increases the risk of acute cardiovascular events. These findings underline the importance of integrated cardiovascular monitoring and early intervention alongside anti-inflammatory treatment to maximize patient outcomes.

## Figures and Tables

**Table 1:** Baseline characteristics of the included patients.

Variable	*n* = 48
Age (Years)	56.83
Female/male ratio	4.3:1
Current smoking, *n* (%)	8 (17%)
Raised BMI (kg/m^2^), *n* (%)	36 (75%)
Arterial hypertension, *n* (%)	28 (58%)
Dyslipidemia, *n* (%)	17 (35.4%)
DM, *n* (%)	9 (19%)
PAD, *n* (%)	0
History of stroke, *n* (%)	1 (2%)
History of MI, *n* (%)	0
Positive RF, *n* (%)	35 (76%)
Positive ACPA, *n* (%)	36 (78%)
JAKi therapy	
Baricitinib, *n* (%)	20 (42%)
Upadacitinib, *n* (%)	15 (31%)
Tofacitinib, *n* (%)	13 (27%)
Concomitant csDMARDs	
Methotrexate, *n* (%)	21 (44%)
Leflunomide, *n* (%)	16 (33%)
Sulfasalazine, *n* (%)	1 (2%)

**Table 2:** Multivariate regression analysis for predictors of cIMT.

Predictor		Coefficient (T0)		Coefficient (T1)		*p* (T0)		*p* (T1)	
**TC**		−0.0014		−0.0010		0.790		0.570	
**LDL-C**		0.0026		−0.0022		0.648		0.318	
**Triglyceride**		−0.0019		0.0006		0.279		0.653	
**Lp(a)**		0.0002		0.0005		0.883		0.581	
**CRP**		−0.0052		−0.1029		0.875		0.105	
**IL-6**		−0.0004		0.0043		0.763		0.369	
**IL-1β**		0.00418		−0.0226		0.476		0.690	
**R**		**R^2^**		**Adjusted R^2^**		**Standard Error of the Estimate**		**Durbin-Watson**	
**T0**	**T1**	**T0**	**T1**	**T0**	**T1**	**T0**	**T1**	**T0**	**T1**
−0.441	−0.569	0.194	0.324	0.003	0.164	0.459	0.306	2.07	2.17
**cIMT**									

**cIMT**: None of the assessed predictors showed statistically significant associations with cIMT at either T0 or T1. The most notable trend was observed for CRP at 12-month follow-up. However, it was not statistically significant. Although a possible inverse association was suggested, statistical significance was not reached. The multivariate linear regression models for cIMT at follow-up demonstrated modest explanatory power, suggesting that the included predictors are accountable for a small proportion of the variability.

**Table 3:** Multivariate regression analysis for predictors of ABI.

Predictor		Coefficient (T0)		Coefficient (T1)		*p* (T0)		*p* (T1)	
**TC**		0.0016		−0.0010		0.673		0.409	
**LDL-C**		−0.0014		−0.00004		0.728		0.976	
**Triglyceride**		−0.0008		−0.0001		0.523		0.873	
**Lp(a)**		0.0002		−0.00004		0.836		0.939	
**CRP**		0.0221		−0.0265		0.346		0.520	
**IL-6**		−0.0001		−0.0014		0.887		0.669	
**IL-1β**		0.0783		−0.0039		0.061		0.917	
**R**		**R^2^**		**Adjusted R^2^**		**Standard Error of the Estimate**		**Durbin-Watson**	
**T0**	**T1**	**T0**	**T1**	**T0**	**T1**	**T0**	**T1**	**T0**	**T1**
0.469	−0.362	0.220	0.131	0.035	−0.075	0.328	0.209	1.85	2.22
**ABI**									

**ABI**: Similar to cIMT, none of the predictors demonstrated statistically significant associations with ABI at either time point. IL-1β at baseline approached statistical significance, indicating a potential, but unconfirmed link with peripheral arterial function. No lipid or inflammatory markers were significantly associated with ABI at baseline or 12-month follow-up.

**Table 4:** Multivariate regression analysis for predictors of carotid plaque presence.

Predictor	Coefficient (T0)	Coefficient (T1)	*p* (T0)	*p* (T1)
**TC**	−0.0027	−0.0015	0.557	0.575
**LDL-C**	0.0016	−0.0017	0.755	0.593
**Triglyceride**	0.0002	0.0009	0.910	0.653
**Lp(a)**	0.0030	0.0037	**0.011**	**0.005**
**CRP**	−0.0356	−0.0691	0.221	0.459
**IL-6**	−0.0019	−0.0048	0.116	0.485
**IL-1β**	0.0240	0.0471	0.657	0.564
**Smoking**	0.3218	0.1675	**0.081**	0.370
**Hypertension**	0.3463	0.1486	**0.014**	0.335
**Pseudo-R^2^ (McFadden)**		**Standard Error**		
**T0**	**T1**	**T0**	**T1**	
0.444	0.325	0.971	1.087	
**Atheromatous carotide plaque presence**				

**Carotid plaque presence:** Among all variables, Lp(a) emerged as the only consistent and statistically significant predictor of carotid plaque presence. Elevated Lp(a) levels were positively associated with plaque presence at both T0 and T1, suggesting a stable and robust relationship. Additionally, arterial hypertension was significantly associated with plaque presence at baseline, though this association was not maintained at follow-up. Smoking showed a trend toward significance at baseline, indicating a possible relationship, although statistically inconclusive.

**Table 5:** Differential analysis of vascular parameters before and after JAKi therapy (T0 vs. T1).

Parameter	Mean at T0	Mean at T1	T1-T0	*p*
**cIMT**	0.29	0.125	−0.165	0.019
**ABI**	0.125	0.04	−0.085	0.103
**Carotid plaque**	0.39	0.47	0.08	0.159

## Data Availability

The original contributions presented in this study are included in the article. Further inquiries can be directed to the corresponding author(s).
